# Needle aspiration of calcific deposits versus shock wave therapy for conservative therapy resistant calcifying tendinitis of the shoulder: protocol of a randomized, controlled trial

**DOI:** 10.1186/s12891-022-05259-z

**Published:** 2022-03-31

**Authors:** Freek Verstraelen, Stéphanie Verhagen, Anouk Giesberts, Inge Bonneux, Henk Koot, Willem den Boer, Marieke van der Steen

**Affiliations:** 1 Department of Orthopaedic Surgery & Trauma, Máxima MC, PO box 5600 PD, Eindhoven, the Netherlands; 2 Department of Orthopaedic Surgery & Trauma, Catharina Hospital Eindhoven, PO box 1350, 5602 ZA Eindhoven, The Netherlands

**Keywords:** Calcific tendinitis, Shoulder, NACD, Barbotage, ESWT, Shock wave therapy, Cost-effectiveness

## Abstract

**Background:**

Calcific tendinitis of the shoulder (CT) is a common disorder with a large disease burden. The initial treatment is with conservative measures. However, when this fails the next step treatment remains unclear. Minimal invasive treatment modalities have emerged. Needle aspiration of the calcific deposits (NACD) and extracorporeal shock wave therapy (ESWT) have both shown good clinical results. Nonetheless, in the current orthopedic literature there are not any studies available that compare both the effectiveness and cost-effectiveness of those two treatment modalities. Therefore, our primary objective is to compare the effectiveness of NACD to ESWT. A secondary objective is to compare the cost-effectiveness of both treatment modalities and workability.

**Methods:**

Following a power calculation using the minimal clinical important difference of our primary outcome (Constant-Murley score, CMS) 140 patients will be included in the study. Enrolment is based upon strict inclusion/ exclusion criteria outlined in the Methods section. Participants will be randomized by computer in two groups (e.g. 70 patients will receive NACD and 70 patients will receive ESWT). The NACD treatment will consist of a sonographically guided removal of the calcific deposits and the ESWT treatment will be a focused ESWT. Both treatments will be conducted according to a standardized protocol, as part of care as usual in our hospital. The primary outcome will be the between group differences in functional outcome (measured with the CMS) between baseline and after 12 months follow-up. Secondary outcomes will be questionnaires regarding the clinical outcome (SST) and quality of life (EQ-5D-5L). Furthermore, NRS pain and cost related questionnaires (iPCQ and ProDisQ) will be collected during follow-up after two months, six months and at final follow-up after 12 months.

**Discussion:**

This study will provide more insight regarding treatment for conservative therapy resistant calcific tendinitis of the shoulder by comparing NACD to focused ESWT, which will aid the physician and patient in determining the appropriate treatment plan.

**Trial registration:**

Dutch trial register: NTR7093 registered on 11 March 2018.

## Background

Calcifying tendinitis (CT) of the shoulder is a common disease of the rotator cuff in which calcium particles are deposited in one or more tendons of the rotator cuff. This can result in a typical pattern of pain, impairments in daily living and decreased range of motion. This disease mainly affects individuals between 30 and 60 years of age and females are more often affected by this condition [1–6]. The etiology of CT of the shoulder is still a matter of dispute. Several hypothesis have been postulated including a degenerative hypothesis, repetitive microtrauma, tenocyte necrosis, reactive and endochondral ossification. All leading to a postulated same end point in which that a locally decreased oxygen tension or hypoxia initiates the formation of the calcific deposit [2].

Initially, the treatment consists of conservative measures such as anti-inflammatory drugs, ice-therapy, physical therapy and/or corticosteroid injections [2, 7–10]. However, if this conservative treatment fails additional treatments must be considered. Historically, the next step treatment has been a surgical procedure [1]. However, other –less invasive– treatment modalities such as needle aspiration of the calcific deposits (NACD) and focused extracorporeal shockwave therapy (ESWT) have emerged. Over the past years both minimal invasive treatments have proven to be effective therapeutic options [9, 11–13]. NACD showed promising results mainly in non-comparing studies [14]. In addition, ESWT has also been proven to be effective, especially high-energy ESWT [11]. Although in the available orthopedic literature both treatment methods seem to be viable options, evidence comparing both treatment methods is limited [15]. Two randomized controlled trials (RCTs) have evaluated and compared the effectiveness of NACD compared to ESWT. In 2014, a RCT was published in which radial shock wave therapy was compared to NACD [16]. However, radial shock wave therapy have been shown to be less effective than focused ESWT and therefore this comparison different from the current study [13]. In 2020, the most recent RCT was published. Louwerens et al [15] compared a protocol of ESWT with four sessions of high-energy focused ESWT to ultrasound guided NACD. Both studies showed that both treatment modalities were effective in treating calcifying tendinitis of the shoulder with low complication rates. However, both studies used markedly different treatment protocols compared to the current study [15, 16]. Therefore, the exact place of NACD or ESWT in the treatment paradigm of CT is not clear yet [10].

Besides, there is very limited evidence available about the cost-effectiveness of any intervention of CT of the shoulder. As far as we are aware, only Haake et al. [7] published results concerning the cost-effectiveness of the treatment of CT. They found that ESWT costs society €1.750 to € 3.500 as a results of being unfit to work compared to €9.710 to €19.440 after surgical treatment for therapy resistant CT of the shoulder. However, there is no data available about the comparison between the minimal invasive techniques (e.g. NACD vs ESWT) [7].

Therefore, this randomized controlled trial (RCT) has several objectives. The primary objective is to compare the short and midterm effectiveness of NACD and ESWT as treatment options for conservative therapy resistant CT to define a preferable minimal invasive treatment. The hypothesis is superiority of either NACD or ESWT regarding functional outcome after 12 months. The secondary objective is to compare the cost-effectiveness of both minimal invasive techniques.

## Methods

### Study design

**Fig. 1:**
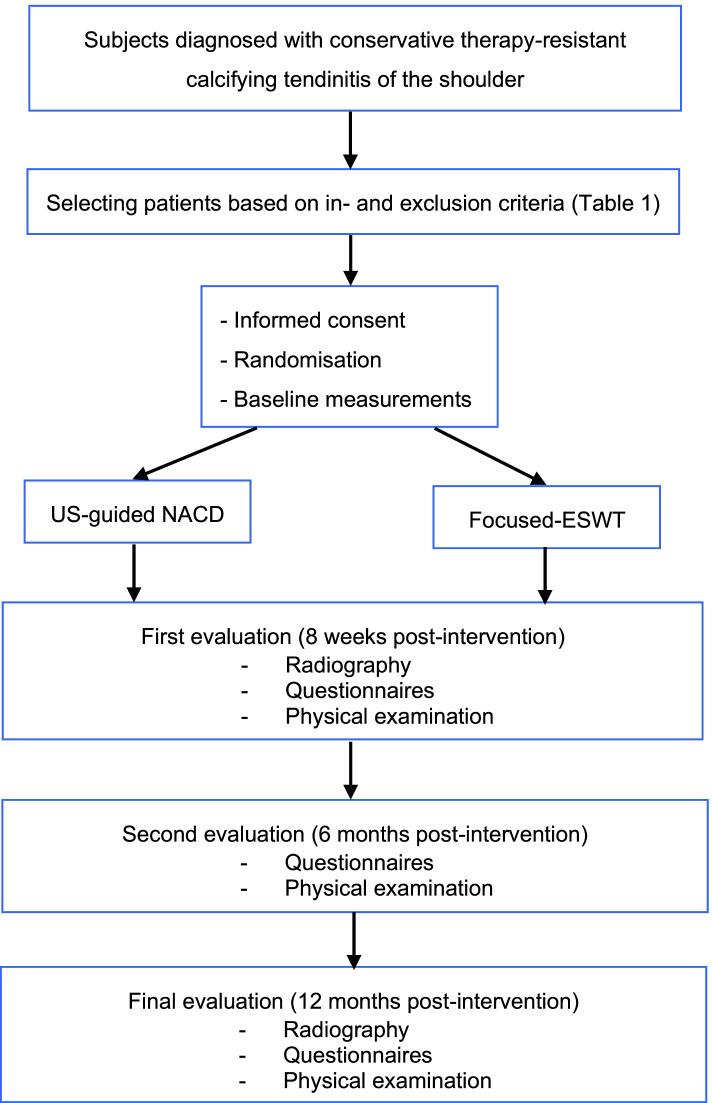
SPIRIT-Flowchart

The design of the current study is a single center open-labeled RCT. Patients will be randomized to receive either NACD or ESWT as treatment for conservative therapy resistant CT of the shoulder (Fig. 1). All patients will be followed for 1 year. The trial was approved by the local medical and ethical commission (METC) on 16^th^ of February in 2018 (NL60762.015.17) and is registered in the Dutch trial register (NL5527/ NTR7093). Amendments were made and approved by the METC.

### Objectives

#### Primary objective

To compare the effectiveness of NACD and ESWT in patients with conservative therapy resistant CT of the shoulder over a period of 12 months. The hypothesis is superiority of either NACD or ESWT in functional recovery based on improvement of the Constant Murley Score (CMS) over a period of 12 months (superiority design).

#### Secondary objectives

1. To assess group differences in change scores on pain and quality of life between baseline and 12 months follow-up, and differences between groups with respect to adverse events and the use of medications in 12 months follow-up.
2. To assess and compare the cost-effectiveness of both interventions over a period of 12 months.

### Setting

The study will be carried out at the outpatient department of orthopedic surgery and trauma of the Máxima Medical Center in the Netherlands. This is a large regional hospital equipped with a training-program for residents in orthopedic surgery. The upper extremity group consists of three experienced orthopedic surgeons.

### Population

Patients with chronic (> 6 months) shoulder complaints, calcifications visible on conventional X-rays and who did not respond to conservative, non-operative therapy for at least three months are eligible for inclusion the study. Further in/exclusion criteria are listed in Table 1.

**Table 1:** In- and exclusion criteria

Inclusion criteria	Exclusion criteria
Age: > 18 years	ESWT or NACD treatment during the last 6 months
Chronic shoulder complaints (> 6 months)	Any contra-indication for the specific treatments (e.g. coagulopathies, malignancies in treated area)
Calcifications visible on conventional radiographs - type I or II calcifications according to the Gärtner classification minimal diameter of 10 mm	Clinical signs of a frozen shoulder or adhesive capsulitis
Able and willing to comply to study protocol	Operations of the affected shoulder in medical history
	Clinical and radiological signs of acute subacromial bursitis
	Full-thickness lesion of the rotator cuff tendon(s) on sonography
	Clinical and radiological sign of acromioclavicular osteoarthritis
	Rheumatoid arthritis or fibromyalgia
	Other intra articular pathology: cartilage lesions, biceps pathology

### Recruitment

All patients are seen and screened for in/exclusion criteria by an experienced shoulder surgeon. If the patient is eligible for inclusion a patient information form is given to the patient and an appointment is made at the specifically for this study created consultation hour. During this consult the eligibility is verified, further information about the study is giving, and after consent the patient will be included and randomized by a researcher. Furthermore, the baseline measurements are assessed and the subject screening and enrollment log filled in.

### Randomization, blinding and treatment allocation

After consent, eligible patients will be randomized and allocated to a treatment group (e.g. either NACD or ESWT) by a computer-generated randomization list using Research Manager (Research Manager, Cloud9 Software, Deventer, The Netherlands). The patients and outcome assessors will not be blinded during the study.

### Treatment of subjects

#### Group NACD

The NACD treatment aims to remove the calcific deposits in the rotator cuff and will be guided by sonography. The procedure will be performed by an experienced musculoskeletal radiologist.

For this procedure the patient will be placed in a supine position with the affected arm towards the radiologist. The skin will be disinfected. Analgesics to the skin, the subacromial bursa and cuff will be administered (Lidocain HydroCloride 1%). Under ultrasound guidance a needle (18 gauge) will be placed in the calcific deposits to fragment these under vacuum. In case of clogged needles during treatment, multiple needles can be used. After the completion of the procedure 40 mg Kenacort will be injected in the subacromial bursa as well under ultrasound guidance.

#### Group ESWT

The ESWT treatment consists of a single session of focused ESWT and aims to fragment the calcific deposits and initiate the resorption phase of calcifying tendinitis. The calcific deposits will be marked sonographically on the patient’s skin with the patient in the exact same sitting position as he/she will receive the focused ESWT. After disinfection of the skin, 1000 pulses focused ESWT with an energy flux density (EFD) of 0.15 mJ/mm^2^ will be applied targeted at the skin marks.

### Rehabilitation after intervention

Both treatment group will receive the rehabilitation as they would have received within the care as usual for CT. This means that after the treatment patients will be instructed to perform pain based movements of the affected arm. The usage of a sling is not standardized. Patients will be instructed to take NSAIDs as needed. Furthermore, if necessary exercise therapy will be prescribed at the consultation two months after treatment. If other co-interventions (such as repeating the treatment procedure, cross-over to other treatment group or even surgical treatment) are necessary during follow-up it will be initiated by the treating orthopedic surgeon.

Intake of painkillers (e.g. NSAIDs), additionally exercise therapy and co-interventions will be recorded in the case report form during study evaluations.

### Baseline characteristics

At baseline several characteristics will be documented, including age, gender, height, weight, body mass index, dominant side, affected side, duration of symptoms, occupations, hobbies. Furthermore, several radiological measurements will be carried out on the conventional X-ray to determine the size of the calcification, Gartner classification [17] and the affected tendon(s).

### Outcome measures

The primary outcome will be the group differences in recovery of functional outcome measured with the Constant-Murley Score (CMS) between baseline and 12 months follow- up. The Constant-Murley score (CMS) is a 100-points scale composed of a number of individual parameters. These parameters define the level of pain and the ability to carry out the normal daily activities of the patient [18]. The Constant-Murley score was introduced to determine the functionality after the treatment of a shoulder injury. The test is divided into four subscales: pain (15 points), activities of daily living (20 points), strength (25 points) and range of motion: forward elevation, external rotation, abduction and internal rotation of the shoulder (40 points). A higher score indicates higher quality of the function [18].

Secondary outcomes measures will be extensive and include the Numeric Rating Scale (NRS) for pain, EQ-5D-5L and simple shoulder test (SST). The NRS assesses pain intensity using a 0–10 ranking scale in which 0 represents “no pain” and 10 “unbearable pain” [19]. EQ-5D is a standardized instrument to measure of health-related quality of life. It consists of the EQ-5D descriptive system and the EQ visual analogue scale (EQ VAS). The descriptive system explores five dimensions: mobility, self-care, usual activities, pain/discomfort and anxiety/depression. The EQ VAS records the patient’s self-rated health on a vertical visual analogue scale, where the endpoints are labelled ‘The best health you can imagine’ and ‘The worst health you can imagine’. The VAS can be used as a quantitative measure of health outcome that reflect the patient’s own judgement [20]. The SST is a patient reported outcome measurement (PROM) in which a score between 0 and 100 can be calculated based on a 12-item long questionnaire [21]. Zero represent the worse shoulder function and 100 a perfect shoulder function. Besides these outcome measures adverse events, co-interventions and co-medications will be monitored during follow-up. Furthermore, the IMTA Productivity Cost Questionnaire (iPCQ) [22] and PROductivity and DISease Questionnaire (PRODISQ) [23] are included to evaluate the cost-effectiveness. The extensive data collection schedule is outlined in Table 2.

**Table 2:** Data collection schedule

**Range**	**Baseline** **T = 0**	**8 weeks** **post-intervention**	**6 months** **post-intervention**	**12 months** **post-intervention**
		± 1 week	± 2 weeks	± 4 weeks
Demographic Information	✓			
Historical records	✓			
Sonography	✓			
HADS	✓			
PCS	✓			
CMS	✓	✓	✓	✓
NRS	✓	✓	✓	✓
EQ-5D	✓	✓	✓	✓
SST	✓	✓	✓	✓
iPCQ	✓	✓	✓	✓
ProDisq	✓	✓	✓	✓
X-rays	✓	✓		✓
Used co-medication		✓	✓	✓
Adverse Events		✓	✓	✓

### Data management

All data will be handled confidentially and is pseudonymized in compliance with the Dutch Personal Data Protection Act. All patient reported outcome measures are collected digitally and all patient data will be stored coded using data management software (Research Manager, Cloud9 Software, Deventer, The Netherlands). Each patient gets a unique study number which is used in all documents regarding the study. Access to the randomization key is restricted to the study-team. The local METC will be annually informed regarding rates of inclusion, adverse events and study results. The local METC graded the study as a ‘low risk’ study. A monitoring plan will be carried out by an external party during execution of the study.

### Statistical analyses

The primary analyses will be performed according to the ‘intention to treat’-principle, indicating that participants will be included in the analysis according to the by randomization allocated group. Secondary analyses will be limited to the compliant participants, independent of which intervention they were randomized (per protocol analysis). Distribution analysis of all variables will be tested by the Shapiro–Wilk test.

Primary outcome will be analyzed by linear regression analyses with change in CMS between baseline and 12 month follow up as dependent variable. The assumptions of constant variance and linear relationships will be assessed using scatter plots. Should any of these assumptions seriously fail then variable transformations will be used. Analyses will be adjusted for baseline variables that change the effect estimate with more than 10%. Similar analyses will be performed for the secondary continuous outcome parameters. Differences between groups on complications and additional treatment will be analyzed by means of Mann–Whitney tests. Furthermore, differences in recovery trajectories between groups will explored by means of mixed ANOVA. Statistical analyses will be performed using SPSS software (IBM, USA). An alpha level of 0.05 will be accepted as significant.

### Sample size calculation

For the sample size calculation, we used the minimal clinically important difference (MCID) for the Constant Murley Score as determined by the study of Kukkonen et al [24].

The reported MCID over a period of 12 months was 10.4 points, with a mean Constant Murray Score of 53.1 (standard deviation of 17.2) at baseline [24].

We aim to find a superiority of one of the two treatment options to treat patients with calcifying tendinitis of the shoulder. Our hypothesis is that superiority of NACD or ESWT above the other treatment in functional recovery over a period of 12 months will be found. Superiority will be expressed by an additional effect size of minimally 0.5. Using a standard deviation of 17.2 of the CMS, we aim to detect a minimally difference of 8.6 points between both groups. Using a power of 80% and an alpha of 0.05 the required sample size is 63 patients per group, resulting in a total of 126 patients. With this number of patients, we will also able to detect a MCID between both groups of 10.4 points (required number of patients is 43 per group). The final sample size required is 140 patients, to accommodate 10% potential dropout rate over 1 year.

## Discussion

This RCT study is the first to compare NACD and a single session of focused ESWT as treatment for conservative therapy resistant calcific tendinitis of the shoulder. The results of this study will aid the physician as it provides valuable information for a shared decision strategy in which treating physician and patient determine the most appropriate treatment. Furthermore, the cost-effectiveness analysis can assist the physician and health care institution to decide whether to provide a certain treatment for conservative therapy resistant calcific tendinitis of the shoulder.

The inclusion period was initially supposed to be approximately two years. In 2015 and 2016 a total of 140 patients with conservative therapy resistant calcific tendinitis of the shoulder were treated with either NACD or ESWT in the Máxima Medical Centre. It was the expected that almost all of these patients would have been eligible candidates for the current study. Although it was expected that 10% of these patients would not be willing to be randomized or comply to the study protocol. Therefore, an inclusion period of two years seemed feasible. However, in the first year the recruitment was slower than expected because more patients than anticipated did not fulfill the in- and exclusion criteria. Therefore, an amendment to the original protocol was made to extend the inclusion period. As a result of the global COVID-19 pandemic, the Orthopaedic outpatient clinic was temporarily closed and the inclusion ceased for a short period. Several attempts were made to speed up the inclusion rate. The first was to include another hospital as participating center for the study. However, this turned out not to be possible because other surrounding hospitals did not offer focused ESWT treatment. The second was to request the department of Rheumatology at the Máxima MC as well as all physical therapists in the region to refer their patients with calcifying tendinitis to the Orthopaedic outpatient clinic for possible inclusion. Inclusion rate are now according to the adjusted feasibility rate. Besides slowing down recruitment, COVID-19 also influenced the follow-up. Patients were not allowed to visit the Orthopaedic outpatient department. As a result, the follow-up of a small number of patients (*n* = 12) was administrated by phone call or video call. This was especially challenging when determining the items abduction, anteflexion and force of the CMS, which requires specific physical examination and testing. In order to obtain the necessary information as reliable as possible one single outcome assessor gave specific instructions to the patient, mainly by explaining the patient how range of motion is determined. Although, the contribution of these items to the total score of the CMS (45 out of 100 points) is considerable in one patient. One should take into consideration that only a small amount of the measurements was done in this manner and they were equally divided between the two groups (seven in NACD group and five in ESWT group). Therefore, the research team expects that potential bias due to this digital compared to in person assessment of the CMS was negligible. Furthermore, the number of patients in which digital assessment was performed was equally distributed between both groups.

Considering the extended inclusion period, additional outcome assessors joined the research team during the execution of the study. The primary investigator trained these assessors to make sure that all measurements were done according to the protocol. In addition, the principal investigator observed the execution of several measurements and give feedback in order to minimize variability in the assessment of the CMS, a measurement which showed good inter-observer reliability in the past [19]. Radiologically assessment will be performed by the principle investigator. In addition, all sessions of focused ESWT treatment will be performed by the same experienced health care professional. Multiple radiologists will perform the NACD treatment, all having a comparable and extensive experience in sonographically guided interventions. Health care professionals performing the ESWT or NACD treatment will not explicitly be informed that the patient is participating in the current study. As a result, it is expected that all performed treatments will be of a comparable high standard.

Despite these precautions, a potential source of bias in the current study might be information bias. Considering the content and logistics of the treatments under investigation, patients and outcome assessors cannot be blinded for treatment allocation. However, since patients at the start of the study are informed that it is not yet known which treatment will lead to the best results and most likely only patients without a pre-existing treatment preference will participate in a RCT, this potential source of bias seems to be small. In addition, outcome assessors will not be the referring shoulder surgeon of the participating patients.

Overall this study will provide more insight regarding treatment for conservative therapy resistant calcific tendinitis of the shoulder by comparing NACD directly to focused ESWT, which will aid the physician and patient in determining the appropriate treatment plan. At time of manuscript submission 118 participants had joined the trial. Current protocol version is 6. The first patient was included in May 2018; the aim is to fulfill the inclusion in 2022 after which the study will be finalized in 2023.

## Data Availability

The datasets generated during and/or analyzed during the current study are available from the corresponding author (freekverstraelen@hotmail.com) on reasonable request.

## Abbreviations

CT: Calcific tendinitis of the shoulder
NACD: Needle aspiration of the calcific deposits
ESWT: Extracorporeal shock wave therapy
CMS: Constant-Murley Score
MCID: Minimal clinically important difference
EFD: Energy flux density
HADS: Hospital Anxiety and Depression Scale
PCS: Pain Catastrophizing Scale
NRS: Numerous rating scale for pain
EQ-5D: EuroQol 5D
SST: Simple Shoulder Test
iPCQ: IMTA Productivity Cost Questionnaire
ProDisQ: PROductivity and DISease Questionnaire
